# Ecologically different earthworm species are the driving force of microbial hotspots influencing Pb uptake by the leafy vegetable *Brassica campestris*

**DOI:** 10.3389/fmicb.2023.1240707

**Published:** 2023-10-04

**Authors:** Cevin Tibihenda, Hesen Zhong, Kexue Liu, Jun Dai, Xiaoqin Lin, Mikael Motelica-Heino, Shuyu Hou, Menghao Zhang, Ying Lu, Ling Xiao, Chi Zhang

**Affiliations:** ^1^College of Natural Resources and Environment, South China Agricultural University, Guangzhou, China; ^2^Tanzania Agricultural Research Institute, Dodoma, Tanzania; ^3^School of Resources and Planning, Guangzhou Xinhua University, Guangzhou, China; ^4^ISTO, UMR 7327, CNRS-Université D’Orléans, Orléans, France; ^5^Department of Civil and Environmental Engineering, Shantou University, Shantou, China

**Keywords:** earthworm, plant–soil system, Pb accumulation, microbial community structure, enzyme activity

## Abstract

Food chain contamination by soil lead (Pb), beginning with Pb uptake by leafy vegetables, is a threat to food safety and poses a potential risk to human health. This study highlights the importance of two ecologically different earthworm species (the anecic species *Amynthas aspergillum* and the epigeic species *Eisenia fetida*) as the driving force of microbial hotspots to enhance Pb accumulation in the leafy vegetable *Brassica campestris* at different Pb contamination levels (0, 100, 500, and 1,000 mg·kg^−1^). The fingerprints of phospholipid fatty acids (PLFAs) were employed to reveal the microbial mechanism of Pb accumulation involving earthworm–plant interaction, as PLFAs provide a general profile of soil microbial biomass and community structure. The results showed that Gram-positive (G^+^) bacteria dominated the microbial community. At 0 mg·kg^−1^ Pb, the presence of earthworms significantly reduced the total PLFAs. The maximum total of PLFAs was found at 100 mg·kg^−1^ Pb with *E. fetida* inoculation. A significant shift in the bacterial community was observed in the treatments with *E. fetida* inoculation at 500 and 1,000 mg·kg^−1^ Pb, where the G^+^/G^−^ bacteria ratio was significantly decreased compared to no earthworm inoculation. Principal component analysis (PCA) showed that *E. fetida* had a greater effect on soil microbial hotspots than *A. aspergillum*, thus having a greater effect on the Pb uptake by *B. campestris*. Redundancy analysis (RDA) showed that soil microbial biomass and structure explained 43.0% (R^2^ = 0.53) of the total variation in Pb uptake by *B. campestris*, compared to 9.51% of microbial activity. G^−^ bacteria explained 23.2% of the total variation in the Pb uptake by *B. campestris*, significantly higher than the other microbes. The Mantel test showed that microbial properties significantly influenced Pb uptake by *B. campestris* under the driving force of earthworms. *E. fetida* inoculation was favorable for the G^−^ bacterial community, whereas *A. aspergillum* inoculation was favorable for the fungal community. Both microbial communities facilitated the entry of Pb into the vegetable food chain system. This study delivers novel evidence and meaningful insights into how earthworms prime the microbial mechanism of Pb uptake by leafy vegetables by influencing soil microbial biomass and community composition. Comprehensive metagenomics analysis can be employed in future studies to identify the microbial strains promoting Pb migration and develop effective strategies to mitigate Pb contamination in food chains.

## 1. Introduction

As one of the biologically non-essential metals in soil, lead (Pb) is very toxic and easily bioaccumulates in organisms even at low concentrations. Pb pollution in leafy vegetable soils poses a serious threat to food safety (Zhou et al., 2021), as the leafy vegetable food chain is a major pathway of human exposure to soil Pb contamination (Wang et al., 2021; Ibrahim and Selim, 2022). Therefore, it is important to explore the mechanisms underlying Pb accumulation in leafy vegetables to ensure food safety. Chinese cabbage (*Brassica campestris*) is a popular leafy vegetable because of its high nutritional value with rich essential nutrients and metabolites (Keim et al., 2020; Liu et al., 2021) and antioxidant activity (Bhavithra et al., 2021; Wang et al., 2022). Approximately 1.5% of the total agricultural land area is used for vegetable cultivation in China. In 2020, vegetable production in China accounted for 30% of the world’s total production [Food and Agriculture Organization (FAO), 2020]. Investigation of Pb accumulation in leafy vegetables in Pb-contaminated soils can provide valuable insights into the potential risks associated with consuming Pb-contaminated produce and the need for effective soil monitoring and remediation strategies.

Metal bioaccumulation in plants and along the food chain mainly depends on the complex interactions between soil organisms and plants (Wu et al., 2020). Earthworm activities in soil influence Pb bioavailability and plant uptake (Jusselme et al., 2015a). Different earthworm species differ in their ecological strategies (Bottinelli et al., 2020; Huang et al., 2021), leading to different effects on Pb entry into the food chain. Moreover, many soil microbes are well-known to influence Pb bioavailability. These microbes include Pb-solubilizing bacteria, plant growth-promoting rhizobacteria, and microbes whose bioactive metabolites help to alleviate Pb phytotoxicity (Yahaghi et al., 2018; Al-Maliki and Al-Shamary, 2022). Studies showed that soil microbial properties influence metal accumulation in rice grains and brassica leaves (Xiao et al., 2017, 2020b). The effects of earthworms on soil microbial communities have been well-documented (Liu et al., 2019; Ahmed and Al-Mutairi, 2022), and the earthworm–microbe symbiotic relationship influences Pb transformation in soil and accumulation in plants (Das and Osborne, 2018). However, it remains unclear how ecologically different earthworm species influence Pb accumulation in leafy vegetables in relation to soil microbial properties.

Potential prospects for effective Pb accumulation in plants lie in revealing the mechanisms behind the earthworm–microbe interaction since these organisms form a discrete ecological unit and serve as the primary engines for terrestrial biogeochemical processes. Earthworms have a strong influence on soil microbial abundance and community composition, mainly in three ways. First, earthworm activities such as digging and casting change the soil microenvironment. Second, the feeding and grazing activities of earthworm lead to the selection of rapidly growing soil microbes through gut-associated processes. Third, microorganisms are dispersed in the soil after surviving the transit through the earthworm digestive tract (Medina-Sauza et al., 2019; Price-Christenson et al., 2020). Bacteria, actinomycetes (ACT), and fungi may contribute differently to plant Pb accumulation due to their different activity levels, abundances, and compositions (Yu et al., 2020; Mahohi and Raiesi, 2021). Nonetheless, soil enzymes secreted by earthworms and microbes are vital indicators of soil biochemical activities and soil quality (Xu et al., 2021). Recent studies reported increased Pb uptake by plants due to changes in soil microbial activities (Liu Y. et al., 2020; Zhang et al., 2022). Previous research on Pb contamination has generally focused on its effect on soil microbial characteristics such as microbial biomass, activity, and diversity (Stefanowicz et al., 2020; Xiao et al., 2020a). However, it is still unclear how different microbial characteristics influence Pb accumulation in plants. This knowledge gap restricts us from fully understanding the mechanisms underlying the effect of microbial hotspots on the rhizospheric soil and plant root functioning.

This study was focused on the earthworm–microbe interactive effect on Pb accumulation in plants. Two ecologically different earthworm species, including the anecic species *Amynthas aspergillum* and the epigeic species *Eisenia fetida*, were investigated. *E. fetida* is commonly found in organic matter-rich environments such as compost heaps and is known to influence the biogeochemical cycle of heavy metals such as Pb, which has a high affinity for organic matter. *A. aspergillum*, a deep-burrowing earthworm species, may affect microbial activity, Pb bioavailability, and Pb uptake by plants. In this study, we tested the following two hypotheses. (1) Ecologically different earthworm species differ in lifestyle, digestive system, and feeding preference and thereby exert different effects on soil microbial biomass, structure, and activity in Pb-polluted soils. (2) Pb accumulation in plants is driven by earthworm-induced changes in soil microbial community composition and activity. To test these two hypotheses, PLFA biomarkers were identified to provide information about the abundances of bacteria, ACT, and fungi and changes in soil microbial community structure. Additionally, fluorescein diacetate (FDA) hydrolysis was measured to evaluate the overall microbial activity in the soil, and the activities of β-glucosidase (β-glu) and N-acetylglucosaminidase (NAG) were determined to investigate the transformation of carbon (C) and nitrogen (N), respectively. The findings of this study will deepen our understanding of the complex interaction between earthworm ecotypes and soil microbes and its effect on Pb accumulation by leafy vegetables (e.g., *B. campestris*) grown in Pb-contaminated soils. In addition, this study will shed light on the soil microbial mechanism of Pb accumulation in the vegetable food chain.

## 2. Materials and methods

The soil used in this study was collected from the 0–20 cm layer of a fallow vegetable field (23°54′ N, 113°27′ E) in Qingyuan City, Guangdong Province, China. The basic properties of the soil were 6.06, 16.6 g·kg^−1^, 1.30 g·kg^−1^, and 12.8 for soil pH, organic C, total N, and C:N ratio, respectively, and total Pb was 20 mg·kg^−1^. Both *A. aspergillum* and *E. fetida* were purchased from a biofertilizer company in Qingyuan, Guangdong Province, China. The seeds of *B. campestris* were provided by the Guangdong Academy of Agricultural Sciences.

The experimental design was described by Tibihenda et al. (2022). The experiment included three earthworm treatments with four replicates. The soil was spiked with PbCl_2_ to set up four Pb contamination levels (i.e., 0, 100, 500, and 1,000 mg·kg^−1^) for each earthworm treatment and aged for 10 months before the experiment started. The three earthworm treatments were: (i) SP, no earthworm inoculation; (ii) SPA, *A. aspergillum* was inoculated; and (iii) SPE, *E. fetida* was inoculated. Each pot (18.5 cm × 12 cm × 16 cm) was filled with 1 kg of soil and planted with three healthy *B. campestris* seedlings at the same growth stage. For the SPA and SPE treatments, 23 ± 1 g of healthy clitellate adult earthworms, or four *A. aspergillum* (5.73 ± 0.40 g individual^−1^) and approximately 58 *E. fetida* (0.4 ± 0.1 g individual^−1^), were introduced into each pot. Soil moisture in each pot was maintained at field capacity. After 30 days of cultivation, the plants were harvested, washed, and oven-dried at 60°C. The earthworms in each pot were collected, counted, weighed, and put in Petri dishes with moisture filter papers at 25°C to empty their gastrointestinal tracts for 7 days. The soil from each pot was divided into two fractions. One was stored at 4°C for later PLFA analysis, and the other was air-dried and passed through a 0.149-mm sieve for organic C and total N determination or a 2-mm sieve for the determination of enzyme activities and other soil chemical properties.

### 2.1. Laboratory analyses

#### 2.1.1. Determination of enzymatic activities

The colorimetric methods used for the determination of β-glu and NAG activities were based on the detection of p-nitrophenol (PNP), with PNP-β-D-glucopyranoside and PNP-N-acetyl-β-D-glucosaminide as substrates, respectively. The soil was incubated at 37°C, and pH 5.0 citrate buffer was used. The enzyme activities were expressed as micrograms of PNP released per gram of soil per hour under standard conditions (μg PNP·g^−1^ soil·h^−1^). Briefly, 2 g of air-dried soil with 0.5 mL of distilled water was incubated at 25°C for 48 h. After 3 mL of ice water was added and mixed thoroughly, 50 μL of the soil suspension was taken, mixed with 25 μL of buffer and 50 μL of the substrate solution, and incubated on an orbital shaker at 37°C for 1 h. The absorbance of the PNP released was measured using a microplate spectrophotometer reader at 405 nm (Mora et al., 2005).

The overall enzymatic activity in soil was assessed by FDA hydrolysis. Air-dried soil (2 g) was incubated with FDA solution in phosphate buffer (pH 7.0) at 25°C for 48 h. The yellow fluorescein released from FDA hydrolysis was quantified using a microplate spectrophotometer reader at 490 nm (Schnvrer, 1982). FDA hydrolysis activity was expressed as μg Fluo kg^−1^ soil h^−1^.

#### 2.1.2. Determination of soil microbial biomass and community composition

PLFA fingerprints were employed as biomarkers to estimate soil microbial biomass and community composition. The PLFA technique involved the extraction of lipids from the soil and the separation of phospholipids from the lipids. The obtained phospholipids were then methylated to form fatty acid methyl esters, which were quantified by gas chromatography. To evaluate the extraction efficiency, an internal standard fatty acid (19:0) was used, and another fatty acid (10:0) was used as an instrumental standard for the gas chromatography analysis, which was added prior to detection and quantification (Au-Quideau et al., 2016). In this study, 14:0, 15:0, 16:0, 18:0, and i18:0 were used as the signature fatty acids for general bacteria (CB), 18:2ω6c and 18:1ω9c for fungi (F), and 10Me 16:0, 10Me 17:0, and 10Me 18:0 for ACT (Frostegård and Bååth, 1996). G^+^ bacteria were characterized by i14:0, i15:0, a15:0, i16:0, i17:0, and a17:0, whereas G^−^ bacteria were characterized by 16:1ω7c, 18:1ω7c, cy17:0, and cy19:0. The total bacterial biomass (total bacteria) was calculated as the sum of G^+^ bacteria, G^−^ bacteria, and CB bacteria. The ratio of fungal biomass to total bacterial biomass was used to estimate the ratio of fungi to bacteria in the soil (Moore-Kucera and Dick, 2008). The total content of PLFAs (total PLFAs) was determined as soil living microbial biomass and expressed as nmol·g^−1^ soil (Frostegård et al., 1996).

### 2.2. Statistical analysis

Data were processed using the SPSS statistical software (16.0). One-way ANOVA, Duncan’s multiple range test, and *t*-test were used to determine the significant differences in variables such as soil enzyme activities and PLFAs between treatments. Data were presented as mean ± standard deviation, and the significance level was set at a *p*-value of <0.05. Principal component analysis (PCA) was performed using the Ade4 package in R statistical software (4.2.3) to evaluate the relationships between soil microbial attributes in the earthworm–plant system. The vegan package in R was used for redundancy analysis (RDA) to explore the variation in contributions of microbial attributes to Pb bioavailability and accumulation in *B. campestris*. The raw data were standardized to eliminate the effects of different data dimensions. In the RDA, soil microbial attributes were treated as explanatory variables, whereas soil available Pb (DTPA-Pb), plant biomass, and plant Pb (concentration and accumulation) were selected as response variables. The Mantel correlation test was then used to evaluate the strength of the correlations between explanatory variables and response variables and to find out other interactions among them.

## 3. Results

### 3.1. Effects of earthworms on soil microbial biomass and structure in the Pb-contaminated soil

The total soil microbial biomass, which was measured as the total content of PLFAs, ranged from 12.5 to 22.1 nmol·g^−1^ soil across the treatments (Table 1). The maximum biomass was observed at 100 mg·kg^−1^ Pb. At 0 mg·kg^−1^ Pb, the presence of earthworms significantly reduced the total content of PLFAs, by 30.9% and 29.3% in SPE and SPA, respectively (*p* < 0.05). When the soil was contaminated with Pb, the total content of PLFAs in SPE increased significantly, whereas that in SP did not change much, and that in SPA only increased significantly at 100 and 500 mg·kg^−1^ Pb. For the same Pb contamination level, the total content of PLFAs was higher in SPE than in SPA, with the difference being significant at 100 and 1,000 mg·kg^−1^ Pb.

**Table 1:** Effects of earthworm inoculation on the biomasses of soil microbial communities and total PLFAs at different Pb contamination levels.

Soil Pb	TR	G^+^	G^−^	CB	ACT	F	Total bacteria	Total PLFAs		
mg·kg^−1^		n mol·g^−1^ soil							G^+^/G^−^	F/B
0	SP	6.91 ± 1.10aA	4.82 ± 0.74aA	1.53 ± 0.24aA	3.11 ± 0.41aA	1.71 ± 0.29aA	13.3 ± 2.05aA	18.1 ± 2.69aA	1.43 ± 0.08aA	0.13 ± 0.01aA
	SPA	4.39 ± 0.72bB	3.78 ± 0.60abA	1.09 ± 0.17bB	2.15 ± 0.37bB	1.39 ± 0.17abB	9.26 ± 1.29bB	12.8 ± 1.76bB	1.18 ± 0.21bB	0.15 ± 0.01aB
	SPE	4.62 ± 1.05bB	3.37 ± 0.62bB	1.06 ± 0.18bB	2.14 ± 0.58bC	1.36 ± 0.10bB	9.04 ± 1.85bB	12.5 ± 2.50bB	1.37 ± 0.08abA	0.16 ± 0.03aA
100	SP	7.36 ± 0.60abA	4.82 ± 0.50bA	1.67 ± 0.13aA	3.34 ± 0.47abA	1.82 ± 0.08bA	13.9 ± 1.16bA	19.0 ± 1.56bA	1.53 ± 0.10aA	0.13 ± 0.01aA
	SPA	6.79 ± 0.86bA	4.64 ± 0.91bA	1.68 ± 0.22aA	2.95 ± 0.26bA	1.96 ± 0.32abA	13.1 ± 1.70bA	18.0 ± 2.12bA	1.50 ± 0.27aA	0.15 ± 0.01aB
	SPE	8.26 ± 0.70aA	6.02 ± 0.55aA	1.85 ± 0.11aA	3.77 ± 0.26aA	2.18 ± 0.15aA	16.1 ± 1.33aA	22.1 ± 1.73aA	1.37 ± 0.05aA	0.14 ± 0.01aA
500	SP	6.67 ± 1.12aA	4.80 ± 0.87bA	1.53 ± 0.24aA	2.82 ± 0.42aA	1.68 ± 0.29bA	13.0 ± 2.12aA	17.5 ± 2.80aA	1.40 ± 0.15aA	0.13 ± 0.01bA
	SPA	6.83 ± 0.66aA	4.68 ± 0.51bA	1.70 ± 0.18aA	2.90 ± 0.23aA	2.24 ± 0.24aA	13.2 ± 1.24aA	18.3 ± 1.67aA	1.46 ± 0.11aAB	0.17 ± 0.01aA
	SPE	7.25 ± 0.94aA	6.19 ± 0.66aA	1.70 ± 0.18aA	2.97 ± 0.42aB	2.02 ± 0.24abA	15.1 ± 1.66aA	20.1 ± 2.26aA	1.17 ± 0.11bB	0.13 ± 0.01bA
1,000	SP	6.80 ± 0.36aA	5.73 ± 0.63bA	1.64 ± 0.11aA	3.03 ± 0.20aA	1.84 ± 0.10abA	14.2 ± 0.95aA	19.0 ± 1.19aA	1.20 ± 0.12aB	0.13 ± 0.01aA
	SPA	5.39 ± 0.26bB	4.46 ± 0.25cA	1.31 ± 0.06bB	2.39 ± 0.16bA	1.70 ± 0.04bB	11.2 ± 0.42bAB	15.2 ± 0.59bB	1.21 ± 0.09aAB	0.15 ± 0.00aB
	SPE	7.04 ± 0.95aA	7.04 ± 1.07aA	1.71 ± 0.20aA	2.96 ± 0.23aB	2.70 ± 0.94aA	15.8 ± 2.22aA	21.4 ± 2.95aA	1.00 ± 0.03bC	0.17 ± 0.05aA

G^+^, Gram-positive bacteria; G^−^, Gram-negative bacteria; ACT, actinomycetes; F, fungi; CB, general bacteria; total PLFAs, total microbial biomass; G^+^/G^−^, Gram-positive to Gram-negative bacteria ratio; F/B, fungi-to-bacteria ratio. SP, no earthworm inoculation; SPA, *Amynthas aspergillum* was inoculated; SPE, *Eisenia fetida* was inoculated. Different uppercase letters indicate significant differences between different soil Pb levels for the same earthworm treatment at a *p*-value of <0.05; different lowercase letters indicate significant differences between earthworm treatments for the same soil Pb level at a *p*-value of <0.05 (*n* = 4).

Generally, bacterial biomass (G^+^, G^−^, and CB) exhibited the same variation trend as the total content of PLFAs. In contrast, fungal and ACT biomasses showed different variation patterns. At 0 mg·kg^−1^ Pb, the presence of either earthworm species (*A. aspergillum* or *E. fetida*) significantly reduced ACT biomass, whereas only *E. fetida* significantly decreased fungal biomass compared to SP. In the Pb contamination treatments, ACT biomass was lower in SPA than in SPE, and the difference was statistically significant at 100 and 1,000 mg·kg^−1^ Pb. Both earthworm species increased fungal biomass at 100 and 500 mg·kg^−1^ Pb compared to SP. Noticeably, a significant difference between SPE and SPA was found at 1,000 mg·kg^−1^ Pb, where the fungal biomass in SPE was significantly higher (Table 1).

In this study, the proportions of specific soil microbial community biomass in total biomass revealed that the G^+^ bacteria had the highest relative abundance, ranging from 33% to 39%, whereas CB had the lowest relative abundance, ranging from 8% to 9% across the treatments. The relative abundances of the microbial communities followed the order of G^+^ > G^−^ > ACT > F > CB (Table 1).

**Figure 1:**
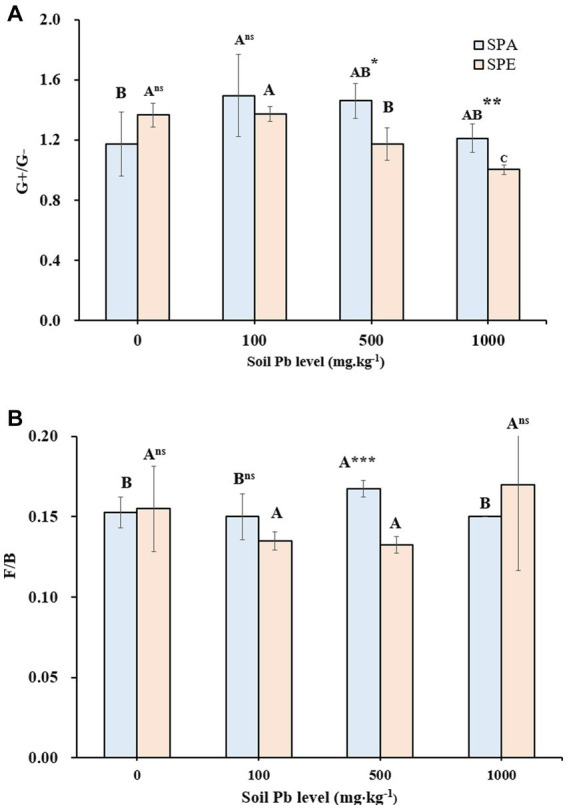
Gram-positive to Gram-negative bacteria ratio (G^+^/G^−^) and fungi-to-bacteria ratio (F/B) in the treatments with earthworm inoculation at different Pb contamination levels **(A)** G^+^/G^−^ ratio **(B)** F/B ratio. SPA, *Amynthas aspergillum* was inoculated; SPE, *Eisenia fetida* was inoculated. Different letters indicate significant differences between different soil Pb levels for the same earthworm treatment at a *p*-value of <0.05. The *t*-test was used to compare the two earthworm treatments for the same soil Pb level, with ^***^p-value < 0.001, ^**^p-value < 0.01, ^*^p-value < 0.05, and ns *p*-value > 0.05 (*n* = 4).

The G^+^/G^−^ ratio ranged from 1.00 to 1.53 across the Pb levels (Table 1), with significantly higher values in SP compared to SPA or SPE except at 100 mg·kg^−1^ Pb. For the SPE treatment, the G^+^/G^−^ ratio decreased with increasing Pb concentration in soil, with significant changes observed at 500 and 1,000 mg·kg^−1^ Pb. In contrast, the G^+^/G^−^ ratio did not change consistently with increasing Pb concentration for the SPA treatment, with a significant difference only observed between 0 and 100 mg·kg^−1^ Pb (*p* < 0.05). The *t*-test showed that the G^+^/G^−^ ratio was significantly higher in SPA than in SPE at 500 and 1,000 mg·kg^−1^ Pb (Figure 1A).

The fungal to bacterial biomass (F/B) ratio ranged from 0.13 to 0.17, with higher values in SPA and SPE than in SP (Table 1). This indicates that the presence of earthworms tended to induce changes in the microbial community, and fungi seemed to benefit more from earthworm activities compared to bacteria. The *t-*test showed that at 500 mg·kg^−1^ Pb, the F/B ratio was significantly higher in SPA than in SPE (Figure 1B).

### 3.2. Effect of earthworms on soil microbial activities in the Pb-contaminated soil

Soil enzyme activities related to the cycling of two biologically essential elements, C and N, were studied at different levels of soil Pb contamination with or without the presence of earthworms. FDA hydrolysis, a measure of overall soil enzymatic activities, ranged from 6.70 to 223.4 μg Fluo kg^−1^ soil h^−1^, with the highest value observed in SP at 0 mg·kg^−1^ Pb and the lowest value also in SP but at 1000 mg·kg^−1^ Pb (Table 2). Generally, as the Pb concentration increased, FDA hydrolysis tended to decrease. For the same Pb contamination level, FDA hydrolysis was generally higher in SPA than in SPE, and the difference was significant at 500 mg·kg^−1^ Pb (*p* < 0.05). In general, the activities of β-glu and NAG were not affected by Pb contamination. NAG activity was stimulated by the presence of *A. aspergillum* at 100 and 500 mg·kg^−1^ Pb and by *E. fetida* at 100 and 1,000 mg·kg^−1^ Pb, whereas β-glu activity was generally inhibited by the presence of earthworms except in SPA at 500 mg·kg^−1^ Pb (Table 2).

**Table 2:** Effects of earthworm inoculation on soil enzyme activities at different Pb contamination levels.

Soil Pb	TR	β-glu	NAG	FDA
mg·kg^−1^		μg PNP·g^−1^ soil·h^−1^		μg Fluo kg^−1^ soil h^−1^
0	SP	905 ± 384aA	452 ± 442aA	223 ± 214aA
	SPA	751 ± 134aB	394 ± 27.3aA	150 ± 161aA
	SPE	632 ± 279aA	237 ± 244aA	51.8 ± 34.1aB
100	SP	639 ± 82.9aA	269 ± 194aA	38.0 ± 44.4bB
	SPA	611 ± 388aB	480 ± 403aA	118 ± 10.4aA
	SPE	565 ± 322aA	277 ± 302aA	128 ± 28.8aA
500	SP	1,179 ± 1093aA	639 ± 1030aA	73.1 ± 27.0abAB
	SPA	1,401 ± 331aA	691 ± 113aA	103 ± 30.0aA
	SPE	946 ± 547aA	390 ± 253aA	50.1 ± 8.42bB
1,000	SP	1,014 ± 150aA	656 ± 538aA	6.70 ± 11.1aB
	SPA	801 ± 321aB	352 ± 281aA	46.8 ± 45.2aA
	SPE	521 ± 558aA	887 ± 812aA	34.0 ± 27.0aB

β-glu, β-glucosidase; NAG, N-acetylglucosaminidase; FDA, fluorescein diacetate. SP, no earthworm inoculation; SPA, *Amynthas aspergillum* was inoculated; SPE, *Eisenia fetida* were inoculated. Different uppercase letters indicate significant differences between different Pb levels for the same earthworm treatment at a *p*-value of <0.05; different lowercase letters indicate significant differences between earthworm treatments for the same soil Pb level at a *p*-value of <0.05 (*n* = 4).

### 3.3. PCA of soil microbial attributes in the earthworm–plant system

**Figure 2:**
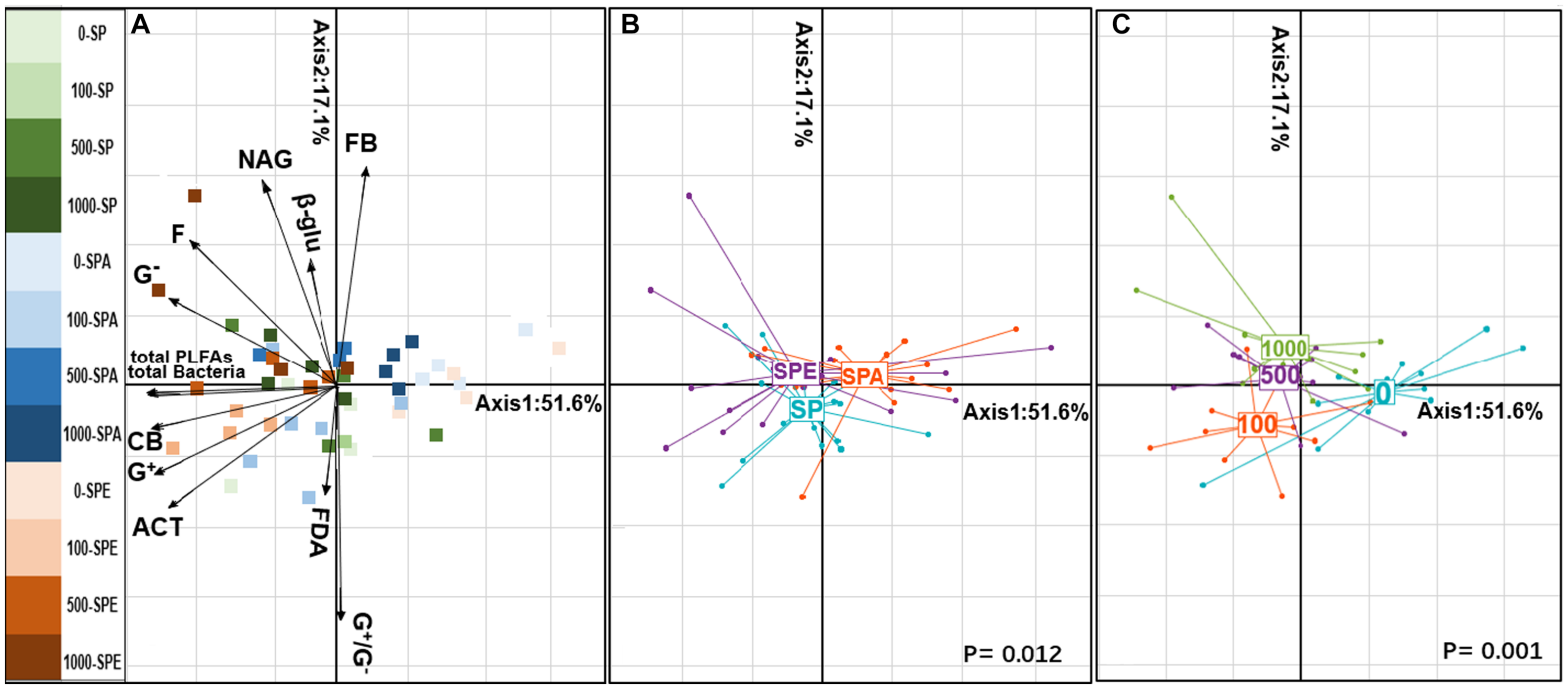
Principal component analysis (PCA) illustrating the distribution of soil microbial attributes in the earthworm–plant system **(A)** PCA projection of soil microbial properties with scattered treatment points. **(B)** Earthworm treatment plot. **(C)** Treatments of different Pb contamination levels of 0, 100, 500, and 1,000 mg kg^−1^ Pb. G^+^, Gram-positive bacteria; G^−^, Gram-negative bacteria; ACT, actinomycetes; F, fungi; CB, general bacteria; total bacteria, total bacterial biomass; and total PLFAs, total microbial biomass; G^+^/G^−^, Gram-positive to Gram-negative bacteria ratio; F/B, fungi-to-bacteria ratio; β-glu, β-glucosidase; NAG, N-acetylglucosaminidase; FDA, fluorescein diacetate. SP, no earthworm inoculation; SPA, *Amynthas aspergillum* was inoculated; SPE, *Eisenia fetida* was inoculated.

PCA analysis revealed a clear separation of the soil microbial attributes between treatments (Figure 2). The first axis, which accounted for 51.6% of the explained inertia, indicated the effects of Pb contamination on soil microbial PLFAs (Figure 2C). Both the total content of PLFAs and the biomass of each microbial consortium were increased in the Pb-contaminated soil with the presence of *E. fetida* (*p* = 0.001). The second axis, representing 17.1% of the explained inertia, described microbial activities and changes in microbial community structure (F/B and G^+^/G^−^; Figure 2B; *p* = 0.012). The presence of *A. aspergillum* led to significant changes in microbial structure and FDA hydrolysis. Importantly, *E. fetida* inoculation was correlated with microbial attributes, suggesting its significant role in influencing soil microbial dynamics. The interspecies comparison of earthworms revealed the different effects of *E. fetida* and *A. aspergillum* on microbial attributes, which in turn affected Pb uptake by *B. campestris* in different ways.

### 3.4. Effect of earthworm activity on the Pb uptake and growth of *Brassica campestris*

The Pb uptake by *B. campestris* in this study was examined by focusing on the following parameters: soil available Pb (DTPA-Pb), plant biomass, and plant Pb (concentration and accumulation). Earthworm inoculation led to a Pb increase in soil availability compared to SP. Different effects on plant biomass were observed, with *E. fetida* promoting and *A. aspergillum* inhibiting *B. campestris* growth. As a result of the higher plant biomass, the SPE treatment exhibited a higher Pb accumulation in *B. campestris* at 100, 500, and 1,000 mg·kg^−1^ Pb. In contrast, the SPA treatment exhibited a higher Pb concentration in *B. campestris*, especially at 500 and 1,000 mg·kg^−1^ Pb (Supplementary Table S1). Earthworm introduction had an effect on the physio-chemical properties of the Pb-contaminated soil, including pH, oxidation–reduction potential (Eh), total N, available N, organic C, dissolved organic C, and cation exchange capacity (Supplementary Table S2). The soil property changes highlighted the significant influence of earthworms on soil biogeochemical processes and Pb migration in the food chain.

### 3.5. Earthworm survival rate in the Pb-contaminated soil

The survival rate of *A. aspergillum* was consistently high, 81.3%, 87.5%, 100%, and 87.5% at 0, 100, 500, and 1,000 mg kg^−1^ Pb, respectively. The survival rates of *E. fetida* were 84.0%, 46.1%, 52.6%, and 62.3% at 0, 100, 500, and 1,000 mg kg^−1^ Pb, respectively. The consistently higher survival rate of *A. aspergillum* at all Pb levels demonstrated the greater tolerance of *A. aspergillum* to Pb contamination compared to *E. fetida* (Tibihenda et al., 2022).

### 3.6. Relationships between soil microbial attributes, plant biomass, and Pb in soil and plant

**Figure 3:**
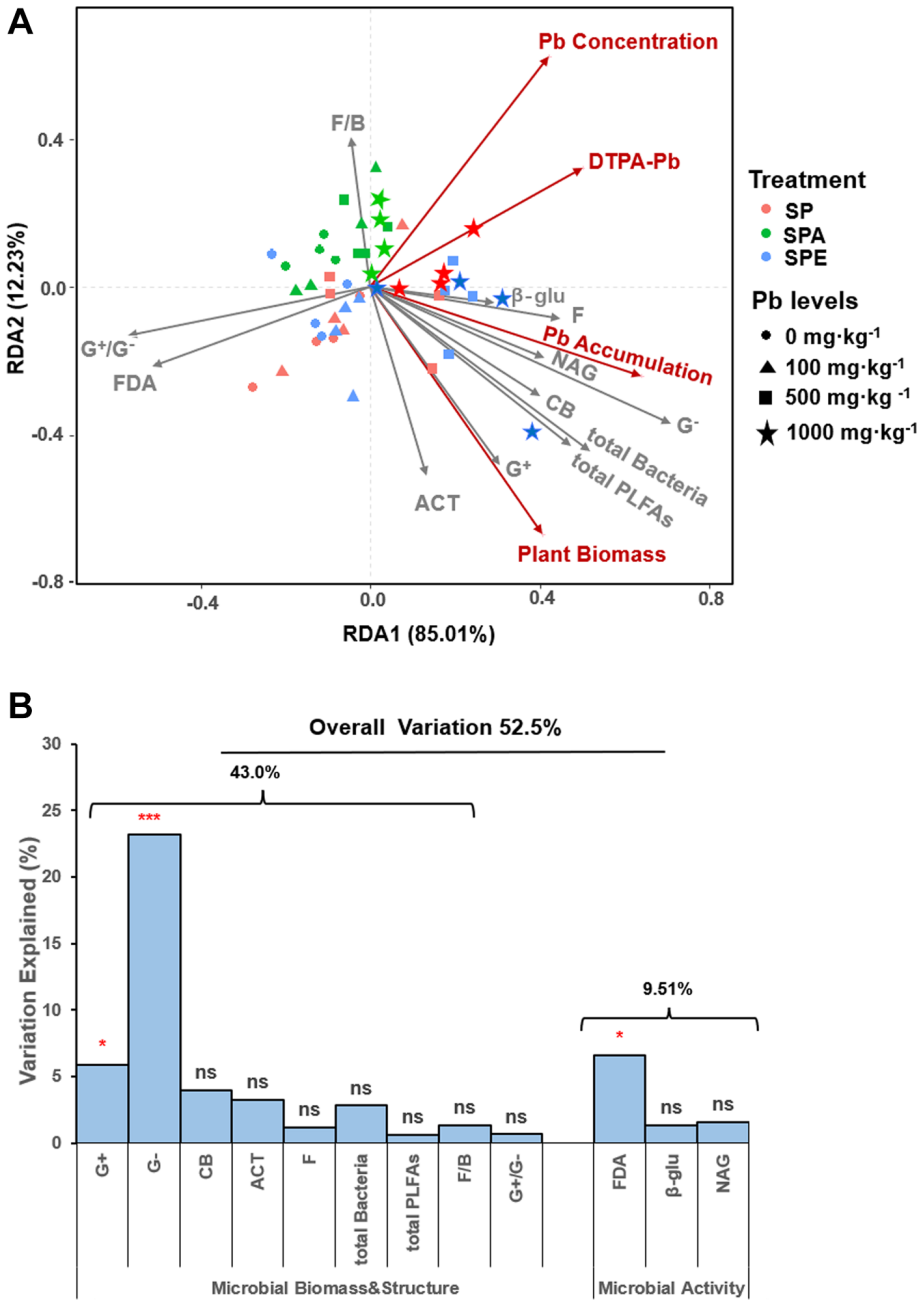
Redundancy analysis (RDA) of soil microbial attributes and plant Pb uptake in the different treatments **(A)** Diagram of RDA of soil microbial attributes as explanatory variables computed over response variables (plant biomass and Pb uptake). **(B)** Contribution of each microbial attribute to the variation in plant biomass and Pb uptake. G^+^, Gram-positive bacteria; G^−^, Gram-negative bacteria; ACT, actinomycetes; F, fungi; CB, general bacteria; total PLFAs, total microbial biomass; total bacteria, total bacterial biomass; G^+^/G^−^, Gram-positive to Gram-negative bacteria ratio; F/B, fungi-to-bacteria ratio; β-glu, β-glucosidase; NAG, N-acetylglucosaminidase; FDA, fluorescein diacetate, SP, no earthworm inoculation; SPA: *A. aspergillum* was inoculated; SPE, *E. fetida* was inoculated; soil Pb contamination levels include 0, 100, 500, and 1,000 mg kg^−1^ Pb. Permutation test was run 999 times to reveal the percentages of variation explained by the explanatory variables, the significance levels indicated by ^***^p-value < 0.001, ^**^p-value < 0.01, ^*^p-value < 0.05, no significant (ns), and *p*-value > 0.05.

The RDA analysis showed that axes 1 and 2 explained 85.0% and 12.2% of the total variation in response variables, respectively (Figure 3A). The soil microbial attributes as explanatory variables significantly explained 52.5% of the variation in response variables (R^2^ = 0.53, Figure 3B). Specifically, soil microbial biomass and community structure explained 43.0% of the observed variation in response variables, whereas microbial activities explained 9.51%. The G^−^ bacteria contributed 23.2% of the total variation in response variables, significantly higher than any other explanatory variable. FDA hydrolysis had a notably high contribution of 6.58%, making it the most prominent microbial activity variable. The ranking order of the top three explanatory variables based on their contributions to the total variation in response variables was as follows: G^−^ bacteria (23.2%, *p* = 0.001^***^) > FDA hydrolysis (6.58%, *p* = 0.019^*^) > G^+^ bacteria (5.90%, *p* = 0.031^*^; Figure 3B). At the high Pb contamination levels (500 and 1,000 mg·kg^−1^), SPE showed strong linear correlations with many explanatory variables and response variables such as plant biomass, DTPA-Pb, and plant Pb accumulation, whereas SPA was correlated with F/B ratio, plant Pb concentration, and DTPA-Pb. In contrast, at low Pb contamination levels (0 and 100 mg·kg^−1^), all earthworm treatments (SP, SPA, and SPE) were associated with FDA hydrolysis and the G^+^/G^−^ ratio. This implies that the increase in the G^+^/G^−^ ratio was negatively correlated with microbial attributes and inhibited Pb uptake.

**Figure 4:**
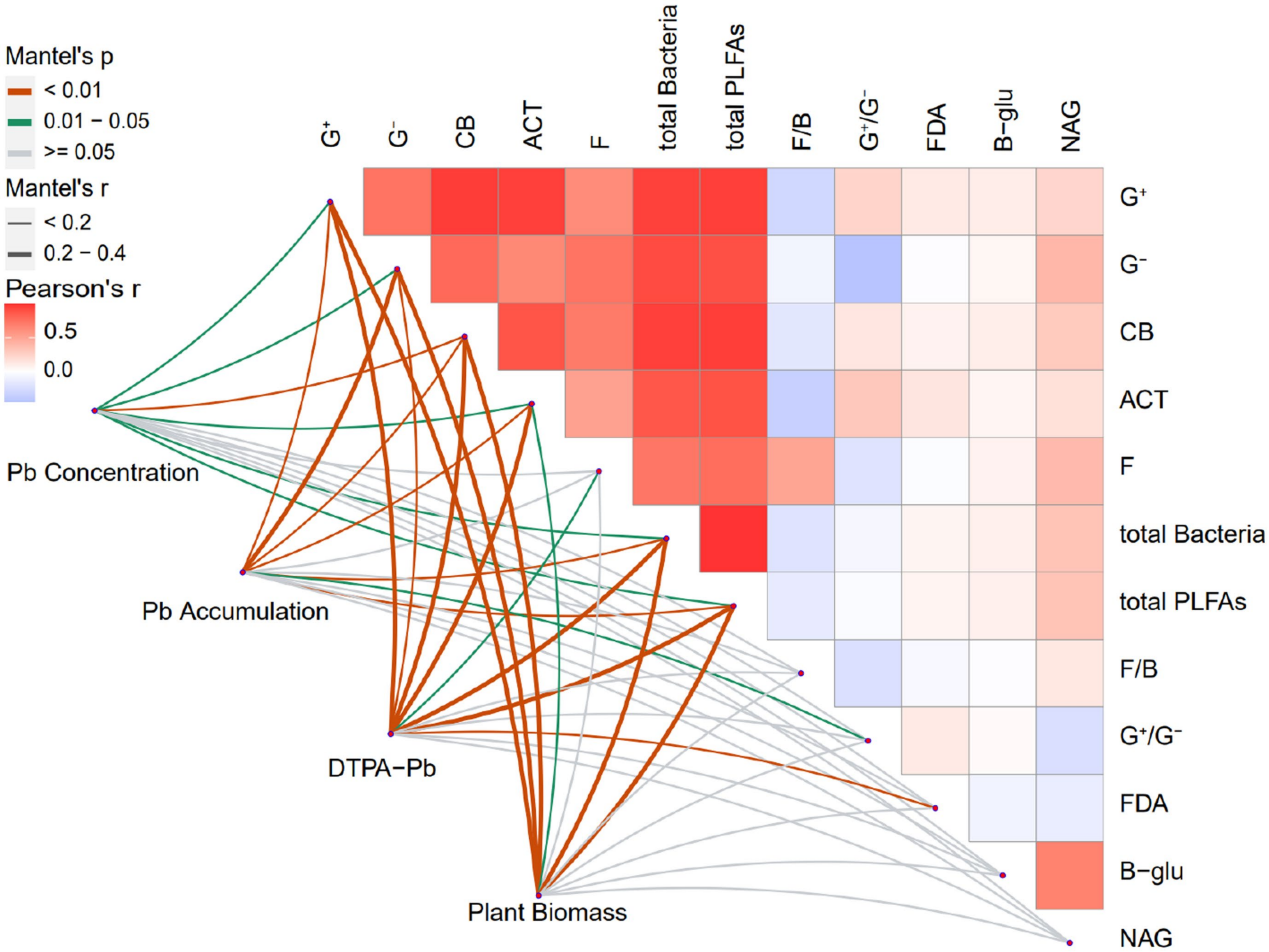
Mantel correlation analysis between soil microbial attributes and Pb uptake indicators in different treatments. G^+^, Gram-positive bacteria; G^−^, Gram-negative bacteria; ACT, actinomycetes; F, fungi; CB, general bacteria; total bacteria, total bacteria biomass; total PLFAs, total microbial biomass; G^+^/G^−^, Gram-positive to Gram-negative bacteria ratio; F/B, fungi to-bacteria ratio; β-glu, β-glucosidase; NAG, N-acetylglucosaminidase; FDA, fluorescein diacetate. SP, no earthworm inoculation; SPA, *Amynthas aspergillum* was inoculated; SPE, *Eisenia fetida* was inoculated. ns, not significant *p*-value >0.05, ^*^p-value < 0.05, ^**^p-value < 0.01, ^***^p-value < 0.001 (*n* = 4).

The Mantel correlation test was utilized to examine the correlations between the explanatory and response variables (Figure 4). The bacterial communities, such as the G^+^ bacteria, G^−^ bacteria, and CB, were significantly positively correlated with DTPA-Pb (*p* < 0.01), plant biomass (*p* < 0.001), Pb accumulation (*p* < 0.01), and Pb concentration (*p* < 0.05). ACT biomass was positively correlated with DTPA-Pb (*p* < 0.001), Pb accumulation (p < 0.01), plant biomass (*p* < 0.05), and Pb concentration (*p* < 0.05). Fungal biomass was positively correlated with DTPA-Pb only (*p* < 0.05). Total PLFAs and total bacteria were significantly positively correlated with all four response variables. Moreover, the G^+^/G^−^ ratio was significantly correlated with Pb accumulation (*p* < 0.05), whereas the F/B ratio did not show a significant effect on the response variables. FDA hydrolysis was significantly correlated with DTPA-Pb (*p* < 0.01). Remarkably, β-glu and NAG activities were significantly correlated with soil microbes. For instance, the G^−^ bacteria, CB, and fungi were correlated with NAG activity. Total PLFAs and total bacteria were significantly correlated with the constituent microbial communities and NAG activity. Some explanatory variables did not contribute significantly to the total variation in response variables but exhibited significant correlations in the Mantel test. These results support the finding of RDA and provide convincing proof that soil microbial attributes, particularly microbial biomass and community, affect Pb uptake by plants with the driving force of earthworms.

## 4. Discussion

### 4.1. Microbial property variations induced by earthworms in the Pb-contaminated soil

The earthworms had a greater influence on the microbial characteristics than on the chemical properties of the Pb-contaminated soil (Supplementary Figure S1). This finding aligns with previous research indicating that the behavioral and feeding traits of earthworms could lead to soil microbial community composition changes as well as soil enzyme activity changes (Omosigho et al., 2022).

At 0 mg·kg^−1^ Pb, the presence of earthworms dramatically reduced the total microbial biomass, which was due to the earthworms feeding on the microbes (Table 1; Edwards and Arancon, 2022). The microbes ingested by earthworms may be killed by the microbicidal substances in the gastrointestinal fluid or may not be able to survive the anaerobic conditions in the earthworm gut (Nechitaylo et al., 2010; Gómez-Brandón et al., 2011; Thakur et al., 2022). Although both G^+^ and G^−^ bacterial biomasses were decreased by earthworm presence (Table 1; Figure 1A), the latter was less negatively influenced. This may be attributed not only to the presence of amino acids, mucosal saccharides, and soluble organic C but also to the neutral pH and anaerobic conditions in the earthworm gut, which are more favorable for the survival and growth of G^−^ bacteria (Liu et al., 2018; Hu et al., 2020; Liu P. et al., 2020).

Soil Pb contamination deleteriously affects enzyme activities, microbial diversity, and microbial abundance (Xiao et al., 2020a; Sun et al., 2023). Pb contamination did not significantly affect the total microbial biomass in SP (Table 1; Figure 2), suggesting that the microbiome in soil may have developed effective Pb resistance and tolerance mechanisms (Mao et al., 2022), resulting in resilience in maintaining microbial biomass under Pb stress. Interestingly, the total microbial biomass was increased in SPE at 100, 500, and 1,000 mg·kg^−1^ Pb (Table 1). This may be because when exposed to high levels of Pb, *E. fetida* secretes a diverse range of substances such as defensive peptides called drilodefensins and other metabolites for Pb detoxification (Gudeta et al., 2023). These metabolites nourish the soil microbes, leading to an increase in total microbial biomass (Table 1). In addition, *E. fetida* prefers high-quality organic matter, which is a good energy source for soil microbes and thereby promotes a high microbial biomass (Lin et al., 2016; Sun et al., 2019; Yadav and Singh, 2023). Another reason for the increased microbial biomass that should not be ignored is the death of *E. fetida* due to Pb toxicity (Tibihenda et al., 2022), leading to less microbes being ingested and killed in the earthworm gut. The higher G^+^/G^−^ ratios in SPA compared to SPE at 100, 500, and 1,000 mg·kg^−1^ Pb indicate that the presence of *A. aspergillum* might be more favorable for the G^+^ bacteria compared to *E. fetida*. The predominance of G^+^ bacteria can also be credited to their exceptional survival in Pb-contaminated environments (Biswas et al., 2021; Borozan et al., 2021). Earthworms generally promote soil fungal and ACT biomasses through their physical and biochemical activities (Medina-Sauza et al., 2019; Song et al., 2020). In contrast, Pb contamination has inhibitory effects on soil fungal and ACT biomasses (Xiao et al., 2020a,b). As a result, the combined effect of earthworm presence and Pb contamination led to complex changes in soil fungal and ACT biomasses.

Microbial activities in soil are biological fingerprints and reliable indicators of Pb toxicity (Luo et al., 2018). In contrast to the study of Yang et al. (2014), this study demonstrated a significant negative correlation between FDA hydrolysis and soil Pb contamination level in SP (Table 2; Figure 2). A high Pb level in the soil would lead to the denaturation of enzymes such as esterases, proteases, and lipases involved in FDA hydrolysis (Patle et al., 2018; Ramana et al., 2021; Li et al., 2022). The different feeding habits of different earthworm ecotypes can have different influences on microbial activity (Boughattas et al., 2019). This may be the reason why the presence of *A. aspergillum* led to a higher FDA hydrolysis activity than that of *E. fetida*. The toxic effect of Pb on soil enzyme activity is primarily due to its binding to the active sites of enzyme proteins (Dick, 1997; Utobo and Tewari, 2015). This suggests that the interface between Pb and enzyme active sites or substrates may be limited even at high soil Pb concentrations, resulting in minimal inhibition of enzyme activities responsible for soil N (NAG) and C (β-glu) transformation. Since a majority of enzymes are released by soil microbes (Wang et al., 2023), the presence and activity of the latter have an important effect on the activities of the former. In this study, soil enzyme activities, notably NAG activity, were enhanced by both earthworm species (Table 2), which was also found in a previous study (Jusselme et al., 2013), indicating the potential of earthworms to stimulate microbial activities in Pb-contaminated soils. In summary, the findings confirm our hypothesis that each earthworm ecotype has a unique effect on soil enzyme activities, microbial biomass, and community composition in Pb-contaminated soils due to its distinct lifestyle and feeding preference.

### 4.2. Influence of soil microorganisms on Pb uptake by *Brassica campestris* with earthworm inoculation

Earthworms influence soil microbial functioning, mitigate the toxic effect of metals on microbial population, structure, and diversity (Šrut et al., 2019; Ren et al., 2021), and promote metal transfer from soil to plant. The RDA results revealed that the soil microbial attributes significantly influenced Pb uptake by *B. campestris*, with a contribution of 52.5%, much higher than the 17% reported by Xiao et al. (2020b). Explaining 43% of the total variation in Pb uptake, soil microbial biomass and community structure had a greater influence on Pb transfer from soil to plant compared to microbial activity (Figure 3B). The increase in soil microbial biomass due to earthworm activities suggests a likely more diverse soil microbial community with a more diverse genetic pool for more diverse soil functions (Tardy et al., 2014; Li et al., 2018) to influence Pb uptake by plants. Pb-accumulating microbes might serve as a potential Pb reservoir for plant Pb uptake (Pham et al., 2022; Rajivgandhi et al., 2022). The G^−^ bacteria in the drilosphere and rhizosphere might play an important role in the Pb uptake by *B. campestris.* They promote Pb uptake under the synergistic effects of plant root exudates and earthworms (Das and Osborne, 2018; WeiXie et al., 2022). G^−^ bacteria release growth regulators, siderophores, exopolysaccharides, and organic acids, which change Pb bioavailability and boost plant growth by reducing Pb phytotoxic effects (Jusselme et al., 2015a; Alexandra et al., 2022). Although G^+^ bacteria were abundant in the Pb-contaminated soil and expected to have a major influence on Pb uptake, they only accounted for 5.90% of the total variation in Pb uptake. The possible reason could be the lack of functioning G^+^ bacterial strains that can influence Pb availability. Earthworm introduction altered fungal abundance, which was positively correlated with soil DTPA-Pb (Figure 4), emphasizing the noteworthy symbiotic colonization of plant roots by fungi to enhance soil Pb bioavailability (Manzoor et al., 2019). The correlations between fungal biomass and enzyme activities (Figure 4) indicate that soil fungi probably secrete enzymes in the rhizosphere and the drilosphere (Frey, 2019). As a result, enzymatic breakdown of organic and inorganic Pb-bearing substrates occurs, and DTPA-Pb increases, which explains the high Pb concentration in *B. campestris* (Supplementary Table S1; Figure 3A). The direct and indirect influences of ACT on Pb uptake by vegetables have rarely been reported. In this study, the earthworms had an effect on ACT biomass, and the latter was correlated with Pb uptake (Figure 4), which was consistent with previous studies (Ali et al., 2017; Liu X. et al., 2020). ACT are well-known to secrete various growth-promoting substances and secondary metabolites, such as metal chelators and organic acids, which promote plant growth and solubilize and mobilize metals, including Pb (Taj and Rajkumar, 2016; Shanthi, 2021). Therefore, ACT played a crucial role in promoting Pb uptake by *B. campestris.* This study provides evidence and new insights into the microbial mechanism of Pb uptake by *B. campestris* driven by earthworms via influencing soil microbial biomass and community structure. Comprehensive metagenomics analysis can be employed in future studies to identify the microbial strains promoting Pb uptake by plants.

The increased Pb accumulation in *B. campestris* was mainly a result of plant growth promotion enhanced by *E. fetida* (Figure 3A). Though biomass increase would lead to Pb concentration dilution (known as the growth dilution effect), the latter would stimulate Pb uptake, eventually resulting in higher Pb accumulation in *B. campestris*. Therefore, earthworms play an important role in promoting metal bioaccumulation and plant tolerance to heavy metal stress in contaminated soils (Mahohi and Raiesi, 2021). The decreased plant biomass in SPA led to higher Pb concentration compared to SPE. Moreover, some studies have demonstrated that plant Pb uptake is related to plant biomass, soil microbial biomass, and community structure (Jusselme et al., 2012, 2015b), which is consistent with our findings in the present study. *E. fetida* played a role in promoting Pb transfer in the vegetable food chain system by decreasing the G^+^/G^**−**^ ratio (Table 1), which was associated with Pb uptake increase (Figure 3A). This finding supports the well-established idea that a change in microbial community structure is always accompanied by a change in microbial activity and community function (Price-Christenson et al., 2020). Furthermore, it was found that SPA exhibited high F/B ratios, DTPA-Pb, and plant Pb concentrations (Figures 1B, 3A). This indicates that *A. aspergillum* introduction tended to shift the microbial community toward being fungi-dominated, which can promote Pb transfer from the soil into the vegetable food chain by improving Pb bioavailability. These findings highlight the potential influence of earthworms on soil ecosystems and the entry of metals into the food chain.

## 5. Conclusion and perspective

Hitherto, studies have provided direct evidence of the cause–effect interaction between earthworms and microbes in the context of Pb accumulation in leafy vegetable systems. However, no study has been conducted to investigate how ecologically different earthworm species differ in promoting the entry of Pb into the food chain. In this study, both soil microbial biomass and community composition had a stronger effect than microbial activity on Pb transfer into the vegetable food chain. The possible soil microbial mechanism of Pb accumulation in *B. campestris* is driven by earthworms, which influence microbial biomass and community structure. In the Pb-polluted soil, the two ecologically different earthworm species exhibited different effects on soil enzyme activities, microbial biomass, and community composition due to their different lifestyles and feeding preferences. However, both earthworm species contributed to Pb uptake by *B. campestris*, which raises a concern about food safety. Understanding the interactions between soil microbes and Pb can help develop effective strategies to reduce Pb contamination in the food chain. Therefore, comprehensive metagenomics analysis can be employed in future studies to identify the microbial strains promoting Pb uptake in vegetable soil systems.

## Data availability statement

The original contributions presented in the study are included in the article/Supplementary material, further inquiries can be directed to the corresponding author.

## Ethics statement

The manuscript presents research on animals that do not require ethical approval for their study.

## Author contributions

CT and HZ: writing-original draft, methodology, formal analysis, and data curation. KL: investigation and funding acquisition. JD: investigation and methodology. XL: data curation and investigation. MM-H: investigation and review and editing. SH and YL: review and editing. MZ and LX: methodology. CZ: conceptualization, methodology, formal analysis, data curation, writing—review and editing, funding acquisition, and project administration. All authors contributed to the article and approved the submitted version.
