# Supplementary material for: Ecologically different earthworm species are the driving force of microbial hotspots influencing Pb uptake by the leafy vegetable Brassica campestris
Source: Front Microbiol. 2023 Oct 4;14:1240707. doi: 10.3389/fmicb.2023.1240707 (PMC10582336; doi:10.3389/fmicb.2023.1240707)
Supplement: Supplementary file 1 [file Data_Sheet_1.pdf]

Supplementary Materials

Table. S1: Effects of Earthworm Inoculation on Pb Uptake and Growth of *Brassica Campestris*

Abbreviations: SP, no earthworm inoculation; SPA, *A. aspergillum* was inoculated; SPE, *E. fetida* was inoculated. Different uppercase letters indicate significant differences between different soil Pb levels for a same earthworm treatment at P < 0.05; different lowercase letters indicate significant differences between earthworm treatments for a same soil Pb level at P < 0.05 (n = 4)

Table. S2: Effects of Earthworm Inoculation and Plant Growth on Soil Chemical Properties under Varied Pb Contamination Conditions

Abbreviations: DOC, dissolved organic carbon; CEC, cation exchange capacity. SP, no earthworm inoculation; SPA, *A. aspergillum* was inoculated; SPE, *E. fetida* was inoculated. Different uppercase letters indicate significant differences between different soil Pb levels for a same earthworm treatment at P < 0.05; different lowercase letters indicate significant differences between earthworm treatments for a same soil Pb level at P < 0.05 (n = 4)

Figure. S1: Principal Component Analysis of Soil Chemical Properties, Microbial Attributes, and Pb Form in Plant and Soil in The Different Treatments

(A) PCA projection of soil chemical–microbial properties and Pb uptake with scattered treatment points

(B) Earthworm treatments plot

(C) Treatments of different Pb contamination levels of 0, 100, 500, and 1000 mg kg^{-1} Pb

Abbreviations: DOC: dissolved organic carbon; G^+, Gram-positive bacteria; G^-, Gram-negative bacteria; ACT, actinomycetes; F, fungi; CB, general bacteria; total bacteria, total bacterial biomass; and total PLFAs, total microbial biomass; G^+/G^-, Gram-positive to Gram-negative bacteria ratio; F/B, fungi to bacteria ratio; β-glu, β-glucosidase; NAG, N-acetylglucosaminidase; FDA, fluorescein diacetate; Pb co, plant Pb concentration; Pb acc, Plant Pb accumulation; SP, no earthworm inoculation; SPA, *A. aspergillum* was inoculated; SPE, *E. fetida* was inoculated.

Table. S1: Effects of Earthworm Inoculation on Pb Uptake and Growth of *Brassica campestris*

Soil Pb	Treatment	Plant Dry Biomass	DTPA-Pb	Pb Concentration	Pb Accumulation
$mg \cdot kg^{-1}$		g	$mg \cdot kg^{-1}$	$mg \cdot kg^{-1}$	$mg \cdot plant^{-1}$
	**SP**	1.48±0.58aB	2.37±0.16bD	19.1±11.7bC	24.5±7.00aB
**0**	**SPA**	0.55±0.25aB	3.00±0.05aD	54.7±32.1aC	26.2±15.1aB
	**SPE**	1.47±1.02aC	2.90±0.27aD	11.3±3.33bB	15.9±9.74aB
	**SP**	1.29±0.26bB	36.4±3.01bC	43.6±22.3aC	53.1±20.2bB
**100**	**SPA**	0.88±0.34bB	35.7±3.99bC	61.8±9.94aC	53.5±19.8bB
	**SPE**	2.20±0.62aBC	41.3±1.56aC	63.6±21.5aB	136±48.3aB
	**SP**	3.50±0.65aA	97.9±4.54bB	170±47.4aB	578±138aB
**500**	**SPA**	1.72±0.38bA	109±2.59aB	257±97.7aB	418±119aA
	**SPE**	3.46±0.81aAB	106±1.58aB	214±89.3aA	759±375aA
	**SP**	3.20±1.91abA	137±2.59aA	352±41.4abA	1133±693aA
**1000**	**SPA**	1.17±0.52bA	139±1.55aA	491±164aA	517±142aA
	**SPE**	4.04±1.13aA	136±1.70aA	294±84.3bA	1189±485aA

Table. S2: Effects of Earthworm Inoculation and Plant Growth on Soil Chemical Properties under Varied Pb Contamination Conditions

Soil Pb mg·kg^{-1}	Treatment	pH	Eh mV	Organic C g·kg^{-1}	DOC mg·kg^{-1}	Total N g·kg^{-1}	Available N mg·kg^{-1}	CEC cmol·kg^{-1}
	SP	6.29±0.07aA	40.8±3.59bB	14.0±0.52aA	75.1±39.0aA	0.69±0.03aA	61.3±6.70aA	3.59±0.18aB
0	SPA	5.82±0.24bA	67.5±13.9aA	14.5±1.38aB	124±60.4aA	0.72±0.05aAB	68.3±6.70aAB	3.66±0.41aA
	SPE	5.62±0.13bB	78.8±7.46aA	14.6±0.66aA	115±19.6aA	0.74±0.05aA	63.0±5.72aA	4.01±0.48aA
	SP	6.37±0.02aA	36.3±1.50cB	12.5±0.89cA	37±12.9bA	0.53±0.02bB	61.3±3.50bA	3.77±0.17aAB
100	SPA	5.91±0.13bA	61.8±7.41bA	17.3±1.11aA	83.5±20.2aA	0.76±0.03aAB	68.3±3.50aAB	3.89±0.21aA
	SPE	5.66±0.15cAB	76.5±8.43aAB	14.3±0.83bA	56.5±28.7abB	0.52±0.02bB	59.5±4.04bA	3.97±0.21aA
	SP	6.27±0.03aA	41.5±1.91bB	14.1±1.11bA	52.5±11.3bA	0.66±0.02cA	66.5±7.00aA	3.89±0.05aA
500	SPA	6.05±0.12abA	54.0±6.83abA	16.4±0.31aAB	113±28.8aA	0.79±0.03aA	70.0±0.00aA	3.98±0.29aA
	SPE	5.92±0.24bA	61.3±13.8B	13.4±2.11bA	47.1±17.4bB	0.72±0.03bA	66.5±9.04aA	3.88±0.19aA
	SP	6.13±0.10aB	49.8±5.50bA	12.9±1.77bA	56.28±10.3bA	0.68±0.07aA	63.0±0.00aA	3.96±0.06aA
1000	SPA	5.88±0.16bA	64.0±9.06aA	14.9±0.92aB	96.6±18.3aA	0.69±0.08aB	63.0±0.00aB	3.81±0.20aA
	SPE	5.89±0.12bA	63.3±6.95aAB	13.2±0.27abA	46.4±12.0bB	0.71±0.04aA	63.0±5.72aA	3.91±0.16aA

Abbreviations: DOC, dissolved organic carbon; CEC, cation exchange capacity. SP, no earthworm inoculation; SPA, *A. aspergillum* was inoculated; SPE, *E. fetida* was inoculated. Different uppercase letters indicate significant differences between different soil Pb levels for a same earthworm treatment at $P < 0.05$; different lowercase letters indicate significant differences between earthworm treatments for a same soil Pb level at $P < 0.05$ ($n = 4$)

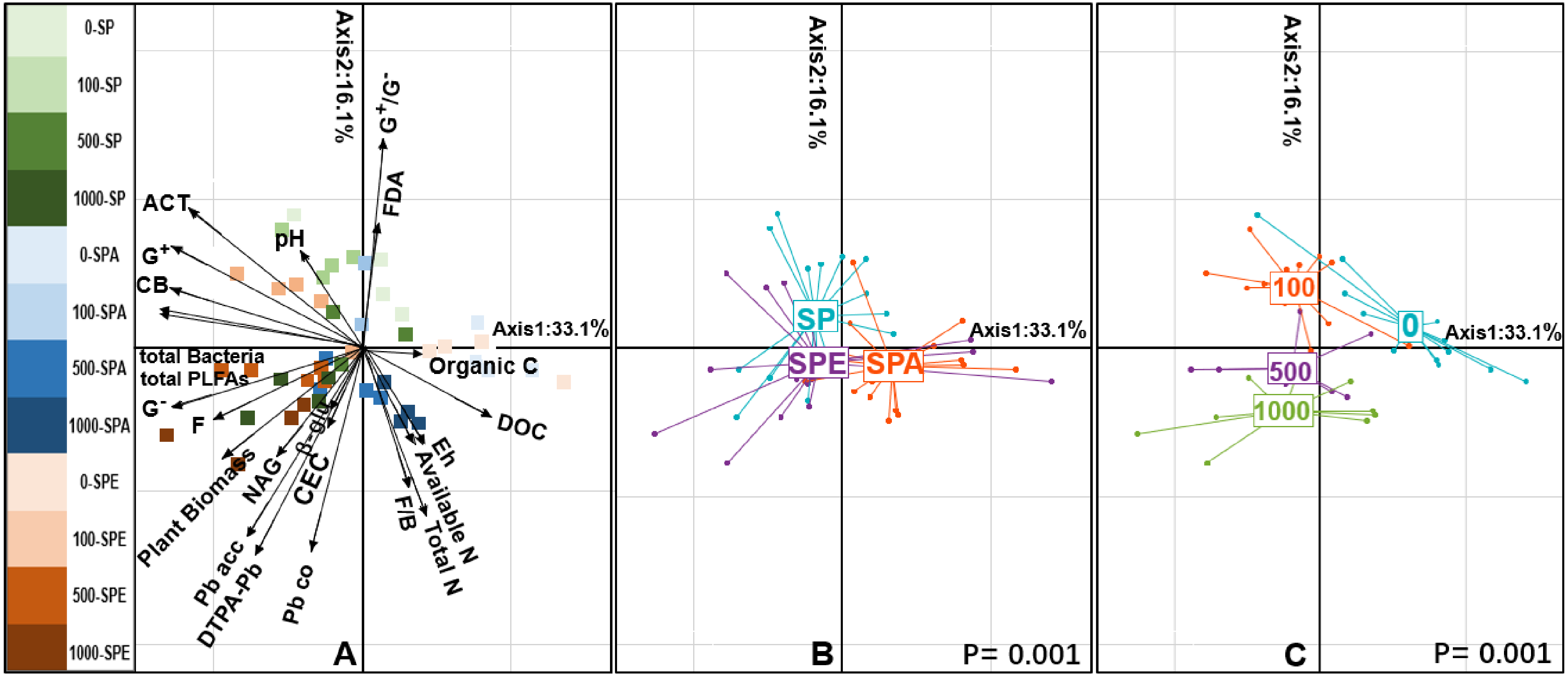

Figure. S1: Principal Component Analysis of Soil Chemical Properties, Microbial Attributes, and Pb Form in Plant and Soil in The Different Treatments

(A) PCA projection of soil chemical–microbial properties and Pb uptake with scattered treatment points

(B) Earthworm treatments plot

(C) Treatments of different Pb contamination levels of 0, 100, 500, and 1000 mg kg^{-1} Pb

Abbreviations: DOC: dissolved organic carbon; G^+, Gram-positive bacteria; G^-, Gram-negative bacteria; ACT, actinomycetes; F, fungi; CB, general bacteria; total bacteria, total bacterial biomass; and total PLFAs, total microbial biomass; G^+/G^-, Gram-positive to Gram-negative bacteria ratio; F/B, fungi to bacteria ratio; β-glu, β-glucosidase; NAG, N-acetylglucosaminidase; FDA, fluorescein diacetate; Pb co, plant Pb concentration; Pb acc, Plant Pb accumulation; SP, no earthworm inoculation; SPA, *A. aspergillum* was inoculated; SPE, *E. fetida* was inoculated.
